# Anomalous Origin and Retropulmonary Course of an Atherosclerotic Stenosed Left Circumflex Coronary Artery

**DOI:** 10.1155/2010/490858

**Published:** 2010-06-13

**Authors:** J. A. T. C. Parker, A. A. Peivandi, S. Bitschnau, N. Kayhan, C. F. Vahl

**Affiliations:** ^1^Department of Cardiothoracic and Vascular Surgery, Hospital of the Johannes Gutenberg-University, Langenbeckstraße 1, 55131 Mainz, Germany; ^2^Department of Radiology, Hospital of the Johannes Gutenberg-University, 55131 Mainz, Germany

## Abstract

We here present the case of a rarely seen anomalous origin and retropulmonary course of the left circumflex artery from the proximal right coronary artery. The patient suffered from coronary ischemia due to stenotic lesions both in the aberrant circumflex coronary artery and in the first and second diagonal branches. Coronary bypass operation was performed.

## 1. Case Description

**Figure 1:**
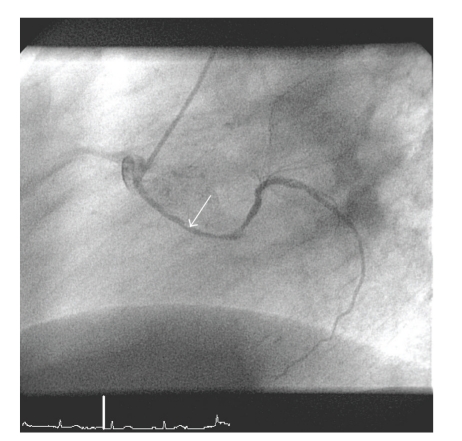
Cardiac catheterization image (LAO) demonstrating the anomalous coronary system. LCx originates from right sinus of valsalva. Mind the retro-aortal route of the LCx (arrow depicts both spiralling course of LCx around the aorta and significant stenosis in LCx).

**Figure 2:**
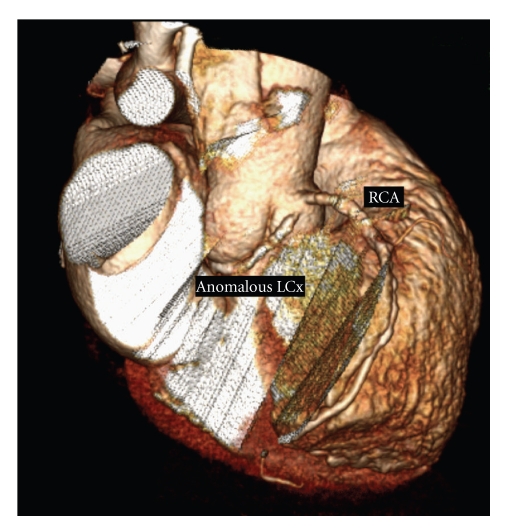
Angio CT showing retro-aortal route of anomalous LCx.

A 66-year-old man with a history of sick-sinus-syndrome, mild arterial hypertension, hyperlipidemia, and carotid atheromatosis was admitted to our hospital with angina pectoris and dyspnea, occurring during exercise (NYHA III) since five months. Physical examination was unremarkable. The ECG showed sinus bradycardia without ST changes. Transthoracic echocardiography showed normal left ventricular systolic and diastolic function with sclerosis of the aortic valve, without insufficiency or stenosis and a low-grade mitral valve insufficiency. Evidence of myocardial ischemia was found when the patient developed ST changes during the treadmill exercise test. Coronary angiography was performed and revealed an anomalous origin of the left circumflex artery (LCx) from the right coronary sinus, in close proximity to the right coronary ostium. In addition, after following a retroaortic course, the LCx spiralled caudal of the pulmonary trunk to resurface in the left atrio-ventricular groove (Figures 1 and 2). The peripheral distribution of the LCx was normal. It showed a significant (90%) obstructive lesion during its course. In addition, second (90%) and third (60%) significant obstructions were detected in the first and second diagonal branches (diagonal (D) first (1), second (2)). Both the left anterior descending coronary artery and the right coronary artery (RCA) showed diffuse sclerosis and normal distribution. Surgical therapy was initiated and we performed a coronary bypass operation with a sequential venous bypass to the LCx and D1.

The postoperative course was uneventful and the patient was discharged 8 days later.

## 2. Discussion

Coronary artery anomalies can be found in approximately 0.6% to 1.3% of all patients who are evaluated with coronary arteriography [1–5]. 

In the case of an anomalous origin of the LCx which is one of the most common anomalies [2], the incidence was 0.34% to 0.48% in large studies by Wilkins and Garg et al. [2, 6]. The coronary origins described in the literature originate from the right sinus of valsalva or from the right coronary artery [2, 6–8]. In our case, the origin of the LCx was from the right sinus of valsalva. Uniquely, it followed a dorsal course around the aorta. 

Dilation of the aortic root and the initial oblique course of the anomalous LCx from the aorta can lead to compression of the coronary artery and shear stress forces on the coronary ostium leading to a narrowed, slitlike configuration of the ostium. In case the proximal segment of the LCx courses behind the aorta, it can form an acute angulation between pulmonary trunk and the left ventricle which can be potentially hazardous. Angulation between the pulmonary trunk and the left ventricle can lead to compression of the intramural segment of the artery. These mechanisms can lead to severe myocardial ischemia, infarction, and even sudden death [2, 9–11]. 

Discussions therefore rebound about the predilection of anomalous coronary arteries for stenotic atherosclerotic lesions [1, 2, 6, 8]. However, due to their low incidence, no study thus far was able to demonstrate convincing evidence for this. In our patient, stenotic lesions were located in the middle segment of the aberrant LCx during its retro-aortal course. This could imply that mechanical compression of the LCx caused by repeated systolic-diastolic movements during the cardiac cycle of the aorta may have contributed to the cause of the stenotic lesion in the aberrant LCx. 

In conclusion, we presented a case of a rarely seen anomalous origin and retro-aortal course of the LCx from the right sinus of valsalva. Both the anomalous LCx and D1 showed significant stenotic lesions and we performed a coronary bypass operation on the symptomatic patient with a sequential venous bypass to the LCx and D1.
